# Approach to Acute Myeloid Leukemia with Increased Eosinophils and Basophils

**DOI:** 10.3390/jcm13030876

**Published:** 2024-02-02

**Authors:** Stavros Papadakis, Ioannis Liapis, Stefanos I. Papadhimitriou, Emmanouil Spanoudakis, Ioannis Kotsianidis, Konstantinos Liapis

**Affiliations:** 1 Department of Hematology, University Hospital of Heraklion, 711 10 Heraklion, Greece; 2 Department of Hematology, Aghios Georgios Hospital, 733 00 Chania, Greece; jonliapis@gmail.com; 3 Department of Laboratory Hematology, Georgios Gennimatas Hospital, 115 27 Athens, Greece; sipapadhimitriou@gmail.com; 4 Department of Hematology, Democritus University of Thrace Medical School, 681 00 Alexandroupolis, Greece; emmanouilspanoudakis@yahoo.com (E.S.); jankots@yahoo.gr (I.K.); kosliapis@hotmail.com (K.L.)

**Keywords:** acute myeloid leukemia, AML, eosinophil, eosinophilia, basophil, basophilia, morphology, CBF, *PDGFRA*, *PDGFRB*

## Abstract

There is remarkable morphologic and genetic heterogeneity in acute myeloid leukemia (AML). In a small percentage of cases of AML, increased eosinophils and/or basophils are present in the bone marrow and sometimes in the peripheral blood. This is often a puzzling diagnostic situation but also an important finding that requires special investigation. Unique chromosomal rearrangements have been correlated with an increased number of eosinophils and basophils in AML. The identification of the underlying genetic lesion that promotes eosinophilia and basophilia can dramatically change both the prognosis and the treatment of the patient. Thus, clinicians must be vigilant in searching for the cause of eosinophilia and basophilia in patients with AML, since the different causes may lead to different treatments and survival outcomes. In this article, we examine the significance of increased eosinophils and/or basophils in the context of AML, provide guidance that simplifies the differential diagnosis, and give prognostic and therapeutic information about specific subtypes of AML associated with eosinophilia and/or basophilia. Evidence supporting personalized (molecularly targeted) therapy for these patients is also presented.

## 1. Introduction

There is remarkable morphologic and genetic heterogeneity in acute myeloid leukemia (AML). Specific chromosomal abnormalities correlate with particular subtypes of AML that have characteristic morphologic and clinical features. Conversely, certain morphologic findings may serve as a basis for the identification of specific genetic subtypes. The presence of eosinophilia and/or basophilia in the context of a myeloid cancer such as AML usually indicates a clonal origin. These eosinophils and basophils are usually part of the neoplastic process, and may be morphologically abnormal with abnormal granulation, abnormal cytochemical reactions or nuclear hyposegmentation. Eosinophilia and/or basophilia are usually found in the bone-marrow aspirates of patients with AML and less frequently in the peripheral blood smears.

Although eosinophilia and/or basophilia may occur as a nonspecific finding in AML, this finding may also indicate specific subtypes of immediate therapeutic and prognostic relevance. Therefore, the presence of increased eosinophils and basophils may be an important diagnostic lead in a patient with AML. The aim of this article is to review the significance of increased eosinophils and basophils in the context of AML, putting emphasis on the appropriate diagnostic evaluation, prognostic significance, and treatment.

## 2. Physiologic Features of Eosinophils and Basophils

### 2.1. Physiologic Features of Eosinophils

Eosinophils are produced in the bone marrow from granulocyte–macrophage colony-forming units (CFU-GM). The latter cells differentiate first into hybrid precursors with properties of basophils and eosinophils (CFU-Bas/Eo) and then into a separate eosinophil lineage. Three cytokines—interleukin-3, interleukin-5, and granulocyte–macrophage colony-stimulating factor (GM-CSF)—are particularly important in the development of eosinophil granulocytes [1,2]. These cytokines are encoded by closely linked genes on chromosome 5q31 and bind to receptors that have a common beta chain (common beta [CD131] subunit) and different alpha chains. The regulation of genes that drive eosinophil differentiation is controlled by a network of transcription factors including C/EBPα, C/EBPε, GATA-1, GATA-2, FOG-1, and PU.1 [2,3]. After maturing in the bone marrow, eosinophils migrate from the circulation into tissues [4,5]. Eosinophils, unlike neutrophils, can survive in tissues for extended periods (perhaps weeks), depending on the cytokines in the microenvironment.

The granules of eosinophils contain a crystalloid core composed of major basic protein (MBP) and a matrix composed of eosinophil cationic protein (ECP), eosinophil peroxidase (EPX), and eosinophil-derived neurotoxin (EDN). In addition, eosinophils produce hydrolytic enzymes such as phospholipase A2 (lysophospholipase), cytokines (some of which are stored in the granules), and large amounts of lipid mediators (leukotriene C4, which is metabolized to leukotriene D4 and E4) that are generated after cellular activation. These three lipid mediators are the slow-reacting substances of anaphylaxis that increase vascular permeability and mucus secretion and are potent stimulators of smooth-muscle contraction [6,7].

Eosinophils normally account for 1–5% of peripheral-blood leukocytes, and the upper limit of the normal range is 0.5 × 10^9^/L. A marked accumulation of eosinophils (>1.5 × 10^9^/L) is referred to as hypereosinophilia. The normal range for eosinophils and their precursors in the bone marrow is 1–5%. Therefore, the cut-off value for increased bone-marrow eosinophilic granulocytes and their precursors is 6%. A definition of severe marrow eosinophilia has been proposed that requires ≥20% of marrow cells to be eosinophils, with or without peripheral-blood eosinophilia [8,9]. There are many causes of reactive (non-clonal) eosinophilia [10].

#### Charcot–Leyden Crystals

**Figure 1:**
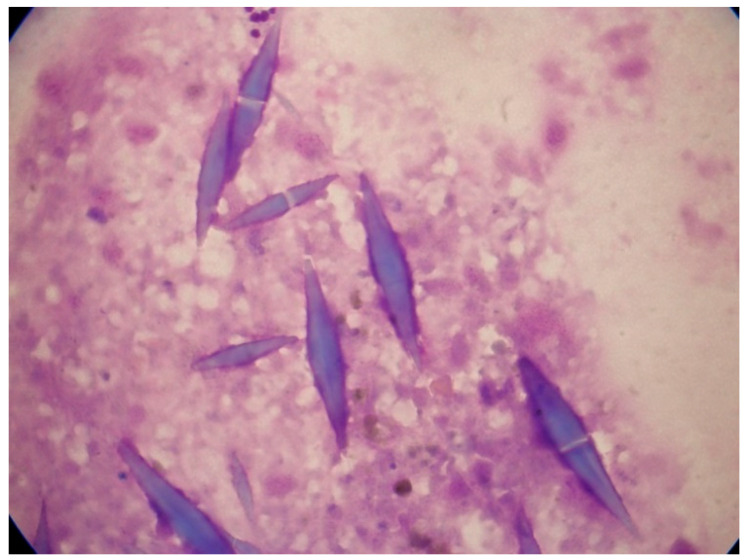
Charcot–Leyden crystals in association with AML. These bizarre bipyramidal crystal structures were noted in the bone marrow aspirate smears of a 75-year-old woman with pancytopenia.

Charcot–Leyden crystals occur in association with marked eosinophilia. Charcot–Leyden crystals were first reported in 1853 by Jean-Martin Charcot, who found tiny crystals in the cardiac blood and spleen of a patient who died from leukemia. In 1872, Ernst Viktor von Leyden also described colorless crystals in the sputum of patients with asthma [11]. Following the discovery of Charcot–Leyden crystals, many conflicting reports appeared as to the chemical nature and significance of these crystals. At that time, it was thought that lysolecithin acylhydrolase, which has lysophospholipase activity, was the constituent protein of Charcot–Leyden crystals. Subsequent investigations revealed galectin-10, a lysophospholipase binding protein, as the constituent protein of Charcot–Leyden crystals. Figure 1 illustrates Charcot–Leyden crystals in association with AML.

### 2.2. Physiologic Features of Basophils

Basophils normally account for ≤2% of peripheral-blood leukocytes, and the upper limit of the absolute basophil count normal range is 0.1 × 10^9^/L. Hyperbasophilia occurs when the peripheral-blood basophil count is >1.0 × 10^9^/L. There are many causes of reactive (non-clonal) basophilia [12]. Persistent basophilia (defined as lasting >8 months) is always suggestive of a neoplastic hematologic disorder. The normal range for basophils and their precursors in the bone marrow is 0–2%.

CD123, commonly expressed in basophils, accounts for the α-subunit of the interleukin-3 receptor (IL-3Rα). Interleukin-3 is the main cytokine for basophilic differentiation and maturation. Other important regulators are GM-CSF, TGF-β, interleukin-5, and thymic stromal lymphopoietin (TSLP) [12]. STAT5 is the effector of interleukin-3 signaling and induces GATA-2-dependent transcriptional activation [13]. Other important transcription factors for basophil development include GATA-1 [14], IRF8 [15], and RUNX1 [16]. Basophils have a short life span (<3 days).

Basophils, like mast cells, carry the high-affinity IgE receptor (FcεRI). Basophils, unlike mast cells, do not contain tryptase. Histamine is the most important constituent of basophilic granules [12]. Their metachromatic granules are also rich in proteoglycans such as chondroitin and heparin and a variety of enzymes. They are also able to produce a variety of growth factors, such as VEGF and HGF, and inflammatory cytokines, namely interleukin-4 and interleukin-13 [17].

#### Metachromatic Granules

The presence of granules taking a metachromatic stain means that they are stained pink or red using blue dyes (toluidine blue or methylene blue). This is due to the large amount of proteoglycans in basophil and mast-cell granules. Toluidine blue staining, which is necessary to reveal the metachromatic character of the granules, stains both the basophilic and mastocytic granules and, therefore, it cannot differentiate between the two [18]. The periodic-acid Schiff stain may aid in the differential diagnosis: basophils typically show a speckled pattern, whereas mast cells exhibit negativity or a very weak reaction [19]. The normal counterpart of the metachromatic blast is thought to be the common progenitor of both the basophilic and mastocytic cell differentiation.

## 3. AML with Increased Eosinophils (Table 1)

### 3.1. AML with inv(16) or t(16;16)

Of the chromosomal abnormalities associated with AML with increased marrow eosinophils (i.e., >5% marrow eosinophils), the best example is inv(16)(p13.1q22) or t(16;16)(p13.1;q22), resulting in the fusion of *CBFB* at 16q22 to *MYH11* at 16p13.1 (*CBFB-MYH11*). *MYH11* encodes for smooth-muscle heavy-chain myosin and *CBFB* codes for the beta subunit of core-binding factor (CBF), a heterodimeric transcription factor that controls the transcription of genes involved in hematopoiesis. The CBFB subunit normally heterodimerizes with RUNX1 (CBFA2), the gene product of *RUNX1,* and increases its DNA-binding affinity. The CBFB-MYH11 mutant protein does not bind RUNX1, leading to RUNX1 sequestration on cytoskeletal structures in the cytoplasm and rendering it inactive [20,21]. The exact mechanism by which these alterations cause eosinophilia remains unclear [22,23].

**Table 1:** Subtypes of AML with increased eosinophils.

AML Subtype	Epidemiology	Clinical Features	Diagnostic Methods	Treatment	Prognosis
AML with inv(16)(p13.1q22)/t(16;16)(p13.1;q22); *CBFB-MYH11*	5–8% of AML cases; more common in young patients	Myelomonocytic (M4) differentiation; abnormal eosinophil morphology (basophilic granules, hypolobated); peripheral eosinophilia uncommon	RT-PCR Karyotype FISH	Standard intensive regimen plus gemtuzumab ozogamicin	Favorable
AML with t(8;21)(q22;q22); *RUNX1-RUNX1T1*	1–5% of AML cases; more common in young patients	Myeloblastic differentiation (M2 or M1); normal eosinophil morphology; excess basophils; peripheral eosinophilia uncommon	RT-PCR Karyotype FISH	Standard intensive regimen plus gemtuzumab ozogamicin	Favorable
AML with t(9;12)(q34;p13); *ETV6-ABL1*	Rare; most common in young men	Typically accompanied by peripheral-blood eosinophilia; abnormal eosinophil morphology (coarse eosinophilic and/or basophilic granules)	FISH RT-PCR Karyotype	Standard chemotherapy plus second-generation TKI	Poor prognosis; aggressive disease
AML with *FIP1L1-PDGFRA*	Most common in young men	Typically accompanied by peripheral-blood eosinophilia; dysplastic eosinophils; frequent mast cells and reticulin fibrosis	FISH RT-PCR	Standard chemotherapy plus imatinib	High rate of complete remission; increased risk of relapse
AML with *PDGFRB* (5q31-q33) rearrangement	Most common in men	Typically accompanied by peripheral-blood eosinophilia (but less prominent than *FIP1L1-PDGFRA*); dysplastic eosinophils	FISH Karyotype	Standard chemotherapy plus imatinib	High rate of complete remission; increased risk of relapse
AML with *FGFR1* (8p11) rearrangement	Wide range of ages (3–84 years)	Frequent hepatosplenomegaly	FISH Karyotype	TKI, midostaurin, pemigatinib	Poor prognosis

Abbreviations: AML, acute myeloid leukemia; FISH, fluorescent in situ hybridization; RT-PCR, reverse-transcriptase–polymerase-chain-reaction; TKI, tyrosine kinase inhibitor.

#### 3.1.1. Morphology

**Figure 2:**
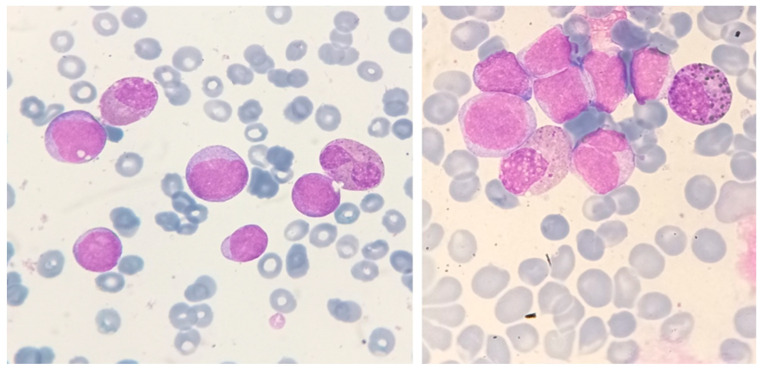
Abnormal eosinophils in the bone-marrow aspirates of a patient with AML with inv(16)(p13.1q22). These micrographs, showing abnormal eosinophils containing both eosinophilic and basophilic staining granules, are representative of myelomonocytic leukemia with eosinophilia (May–Grünwald–Giemsa, ×1000).

AML with inv(16) or t(16;16) is included in the current World Health Organization (WHO) classification of tumors of hematopoietic and lymphoid tissues in the category of “acute myeloid leukemia with defining genetic abnormalities” [24]. It corresponds morphologically to acute myelomonocytic leukemia with eosinophilia (subtype M4Eo, according to the French–American–British [FAB] classification); rarely, cells with these features can be identified as acute myeloblastic leukemia with maturation (M2). The bone marrow usually shows an increased number of eosinophils at all stages of maturation; they usually amount to ≥5% of non-erythroid cells (Figure 2).

Most importantly, these eosinophils are abnormal, and some have, in addition to the characteristic specific eosinophilic granules, large basophilic (immature) granules and may have a single unsegmented nucleus. The basophilic granules are larger and more irregular, as well as more numerous, than those occasionally seen in immature eosinophils. The anomalous immature granules are mostly evident at the late promyelocyte and myelocyte stages; however, they tend to persist in some band and segmented forms. Unlike normal eosinophils, these cells stain with chloroacetate esterase and periodic-acid Schiff. The peripheral blood is not different from that in other cases of acute myelomonocytic leukemia; eosinophils are not increased in the peripheral blood. AML with inv(16)(p13.1q22) or t(16;16)(p13.1;q22) is associated with a high rate of complete remission and a favorable overall survival rate when treated with intensive induction and consolidation therapy.

#### 3.1.2. Confirmation of Diagnosis

**Figure 3:**
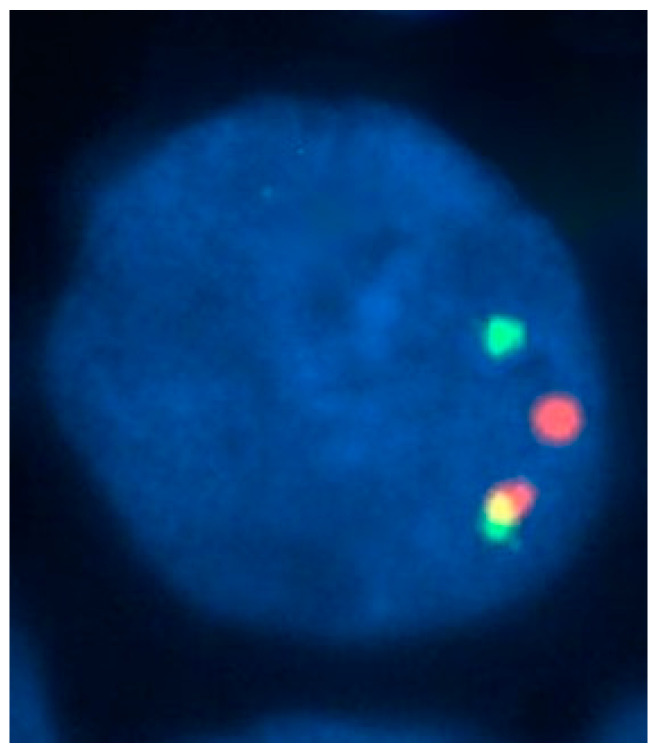
An interphase fluorescence in situ hybridization (FISH) study with a dual-color break-apart probe set containing two probes flanking the breakpoint in the *CBFB* gene, showing a nucleus with inv(16)(p13.1 q22). An intact gene results in the colocalization of the two probes, producing a fusion (yellow) signal. The presence of *CBFB* rearrangement is indicated by separate red and green signals.

By conventional cytogenetic analysis, inv(16)(p13.1q22) is a subtle rearrangement that may be overlooked when metaphase preparations are suboptimal. In addition, occasionally, cytological features of AML with abnormal eosinophils may be present without karyotypic evidence of a chromosome 16 abnormality, but with *CBFB-MYH11* nevertheless demonstrated by molecular studies such as a reverse-transcriptase–polymerase-chain-reaction (RT-PCR) or fluorescent in situ hybridization (FISH), as shown in Figure 3. Therefore, RT-PCR and/or FISH for *CBFB-MYH11* should be requested in AML cases (typically M4) with a proliferation of abnormal eosinophils.

### 3.2. AML with t(8;21)

AML with t(8;21)(q22;q22) translocation may also present with marrow eosinophilia. The t(8;21)(q22;q22.1) involves *RUNX1* (*AML1*), which encodes the alpha subunit of CBF, and *RUNX1T1* (*ETO*): *RUNX1-RUNX1T1* (*AML1-ETO*). The CBF transcription factor is essential for hematopoiesis; transformation by *RUNX1-RUNX1T1* results from the transcriptional repression of normal RUNX1 target genes via the aberrant recruitment of nuclear transcriptional co-repressor complexes. AML with t(8;21) is included in the current WHO classification in the category of “acute myeloid leukemia with defining genetic abnormalities” [24]. Solid tumor manifestations, such as myeloid granulocytic sarcoma, can be present at diagnosis. Although the mechanism of eosinophilia in AML with t(8;21) remains unknown, it has been shown that cultures with leukemic cells harboring t(8;21) can proliferate and differentiate into eosinophils in the presence of IL-5 [25].

#### 3.2.1. Morphology

**Figure 4:**
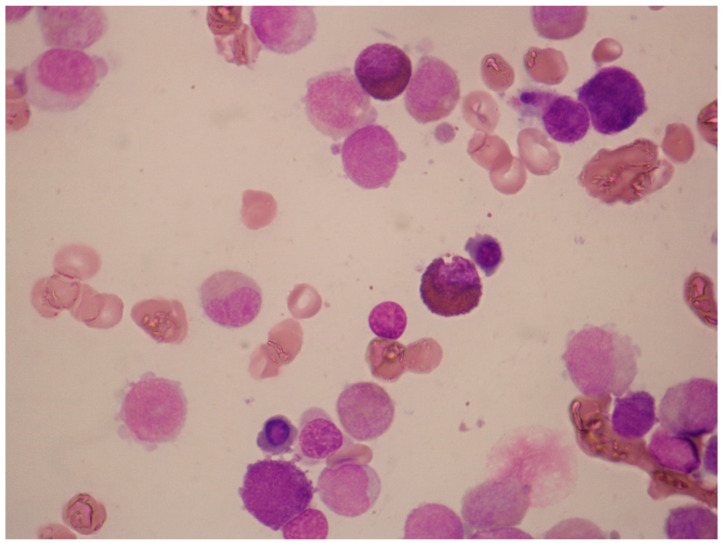
Bone-marrow aspirate smear of a 54-year-old man with AML with t(8;21)(q22;q22) translocation and eosinophilia. The white-cell count was 2.0 × 10^9^/L, without increased eosinophils in the peripheral blood. In the bone marrow, however, eosinophils and eosinophil precursors constituted 22% of cells, without abnormal basophilic granules (May–Grünwald–Giemsa, ×1000).

Most cases of t(8;21) are classified as M2 and rarely as M1 or M4, according to the FAB classification. Eosinophils and eosinophil precursors are frequently increased but they lack the cytological or cytochemical abnormalities seen in AML with inv(16) or t(16;16). Thus, cases of M2 with t(8;21) and marrow eosinophilia lack the abnormal granules seen in M4 with eosinophilia (Figure 4).

An excess of basophils is commonly present in AML with t(8;21). Like AML with inv(16) and t(16;16), eosinophils are not increased in the peripheral blood. AML with t(8;21) is associated with a high complete remission rate and favorable overall survival when treated with intensive induction and consolidation therapy.

#### 3.2.2. Confirmation of Diagnosis

Usually, t(8;21)(q22;q22) is readily detected by means of conventional cytogenetic analysis. RT-PCR and FISH may allow for an earlier detection of *RUNX1-RUNX1T1* in cases of AML with an eosinophilic component without abnormal granules.

### 3.3. AML with ETV6-ABL1 (ΤΕL-ABL1)

The t(9;12)(q34;p13) involves the *ETV6* (*TEL*) gene at 12p13, a transcription factor frequently rearranged in myeloid and lymphoid leukemias, and *ABL1* at 9q34. The *ETV6-ABL1* fusion gene leads to the activation of ABL1 kinase. *ETV6-ABL1* is a notable rearrangement involved in AML with eosinophilia [26,27,28]. AML with *ETV6-ABL1* is included in the current WHO classification in the category of “myeloid/lymphoid neoplasms with eosinophilia and tyrosine kinase gene fusions” [24].

AML with *ETV6-ABL1* is more common in men than women and occurs predominantly between the ages of 20 and 50 years. It generally has an adverse prognosis with a poor response to traditional AML therapy or imatinib [29,30]. Recent reports suggest higher response rates and improved survival outcomes with second-generation tyrosine-kinase-inhibitor therapy, such as dasatinib and nilotinib, followed by allogeneic hematopoietic-cell transplantation [30,31].

#### 3.3.1. Morphology

The *ETV6-ABL1* fusion gene seems to have *BCR-ABL1*-like activity and, therefore, patients usually present with a picture that resembles atypical chronic myeloid leukemia (aCML) with marked peripheral-blood eosinophilia. However, this translocation has also been reported in AML (usually M1 or M2) with peripheral eosinophilia [27,32]. The presence of peripheral eosinophilia and abnormal bone-marrow eosinophils with coarse eosinophilic and/or basophilic granules is the morphologic hallmark of this type of AML.

#### 3.3.2. Confirmation of Diagnosis

The t(9;12)(q34;p13) translocation is very difficult to be identified by conventional cytogenetics since it might result from cryptic translocation or complex chromosomal translocations involving more than two chromosomes at more than two breakpoints, and usually requires FISH with a combination of *ETV6* and *ABL1* probes. However, there are cases in which FISH is not diagnostic (*ETV6-ABL1* occult fusion) and that require targeted RT-PCR or next-generation sequencing techniques (e.g., RNA sequencing [RNAseq] or whole-genome sequencing [WGS] for the detection of cryptic rearrangements) to be identified [27]. Other techniques such as spectral karyotyping (SKY) have also been used to detect abnormalities involving *ETV6* otherwise missed by conventional karyotyping [33,34].

### 3.4. AML with PDGFRA, PDGFRΒ, and FGFR1 Rearrangements

These translocations create fusion tyrosine kinases with ligand-independent tyrosine–kinase activity, leading to uncontrolled cell proliferation and the stimulation of downstream signaling pathways, including those involving phosphatidylinositol 3-kinase and mitogen-activated protein kinases that promote proliferation and survival. The clinical and hematological features are influenced by the partner gene involved. These disorders typically present as chronic myeloproliferative neoplasms with high-grade peripheral eosinophilia, but may also present as de novo AML. Cases of AML with *PDGFRA*, *PDGFRΒ*, and *FGFR1* rearrangements are included in the current WHO classification in the broad category of “myeloid/lymphoid neoplasms with eosinophilia and tyrosine kinase gene fusions” [24].

#### 3.4.1. AML with *PDGFRA* Rearrangements

**Figure 5:**
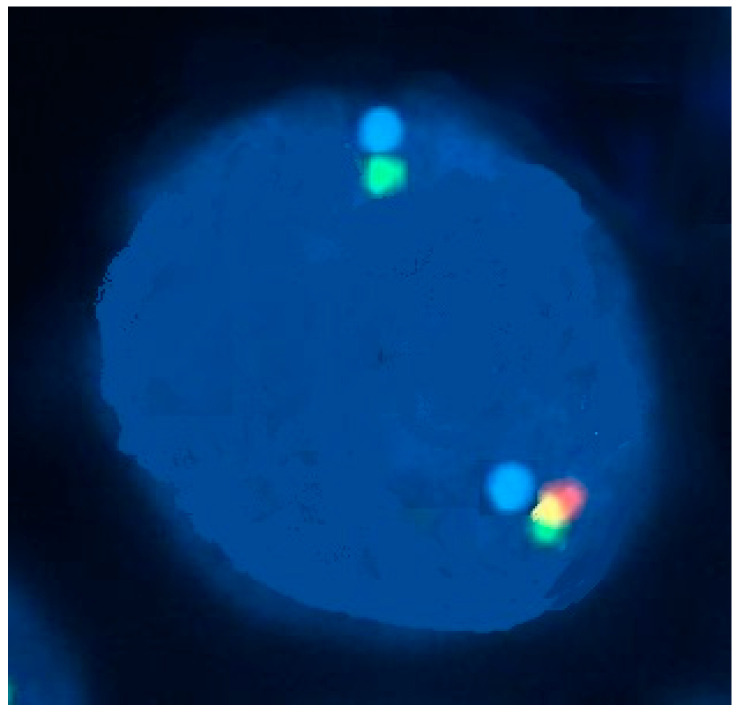
Detection of *FIP1L1-PDGFRA* in a case of AML with eosinophilia, using a three-color probe strategy. On interphase fluorescence in situ hybridization performed with the use of probes to *LNX1* (in red), *FIP1L1* (in green), and *PDGFRA* (in aqua), the nucleus shows one chromosome with all three signals intact and another chromosome with intact *FIP1L1* and *PDGFRA* signals but without the *LNX1* signal (i.e., loss of red signal). This finding indicates a deletion of the region between *FIP1L1* and *PDGFRA* on chromosome 4q12, consistent with the *FIP1L1-PDGFRA* fusion.

*PDGFRA*-related disorders usually present as hypereosinophilic syndrome (HES) of chronic eosinophilic leukemia (CEL). Rarely, they may present as AML with peripheral-blood eosinophilia. The most common abnormality is a fusion of the Fip1-like 1 (*FIP1L1*) gene to the platelet-derived growth factor receptor α (*PDGFRA*) gene generated by an interstitial deletion on chromosome 4q12 between the two genes known as *CHIC2* locus or *LNX* locus, with a resultant juxtaposition of *FIP1L1* and *PDGFRA* [35]. *FIP1L1-PDGFRA* encodes a constitutively activated tyrosine kinase that transforms hematopoietic cells. Because the deletion is small (~800 kb), it is not visible by conventional karyotype and should be sought after by FISH for *CHIC2* or *LNX* deletion, as shown in Figure 5, or RT-PCR for the identification of *FIP1L1-PDGFRA* transcripts. Most patients with *FIP1L1-PDGFRA* are men (male/female ratio, 9:1) [9]. *PDGFRA* translocations may rarely occur with other partners such as *BCR* and *ETV6* [36].

**Figure 6:**
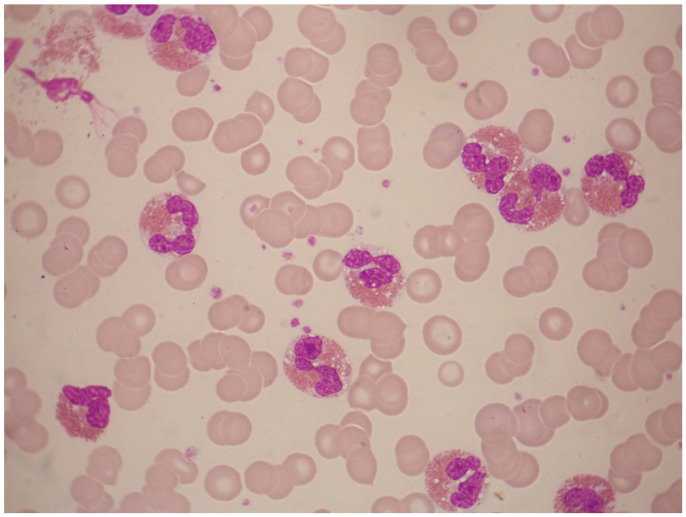
Abnormal eosinophil morphology associated with *FIP1L1-PDGFRA* rearrangement. This peripheral-blood smear shows eosinophils with trilobed nuclei or hypersegmented eosinophils as well as eosinophils with many cytoplasmic vacuoles due to degranulation (May–Grünwald–Giemsa stain, ×1000).

Peripheral-blood morphology is useful in *FIP1L1-PDGFRA* myeloid neoplasms because it may show many abnormal eosinophils [10], including the following:

- Eosinophils with a trilobed nucleus or hypersegmented eosinophils;
- Unilobed eosinophils (nuclear hyposegmentation);
- Eosinophils with reduced or sparse granulation (abnormal granulation);
- Eosinophils with smaller than normal granules (microgranulation);
- Eosinophils with many cytoplasmic vacuoles due to degranulation (Figure 6).

The *FIP1L1-PDGFRA* fusion gene leads to eosinophil proliferation, mediated by the phosphatidylinositol 3-kinase (PI3K), ERK1/2, and STAT5 signaling pathways [37].

#### 3.4.2. AML with *PDGFRB* Rearrangements

**Figure 7:**
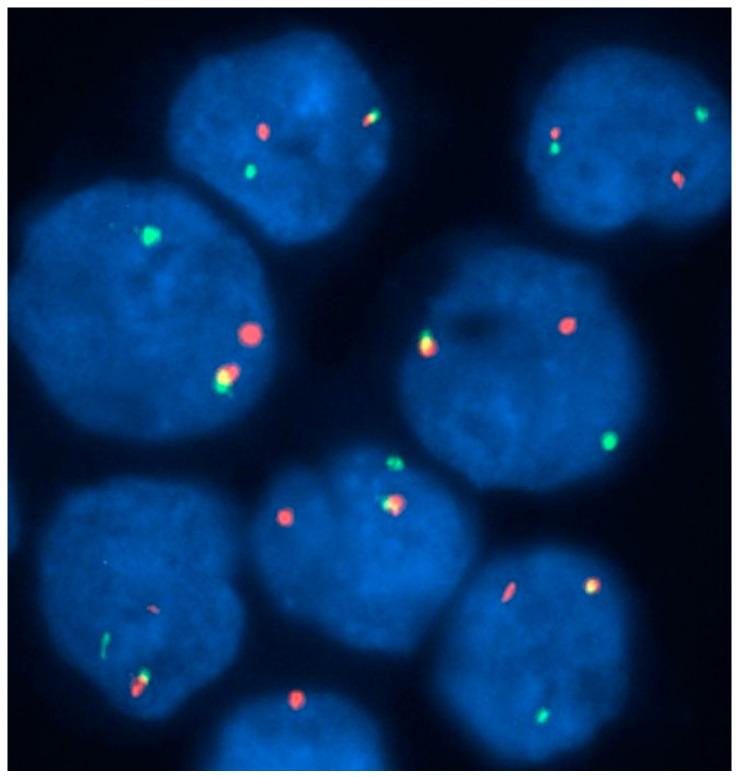
A 70-year-old man with monocytosis and eosinophilia due to *PDGFRB* rearrangement. An interphase fluorescence in situ hybridization study with a dual-color break-apart probe set containing two probes flanking the sequence of *PDGFRB* gene showed that most cells had one fused signal and separate red and green signals, indicating disruption of the *PDGFRB* gene (a normal gene produces a colocalization, i.e., a yellow signal, whereas a rearranged gene results in two separate green and red signals).

Chromosomal translocations involving *PDGFRB* on chromosome 5q33 are much more heterogeneous than *PDFGRA*. Over 30 partner genes for *PDGFRB* have been identified, of which *ETV6* is the commonest, producing *ETV6-PDGFRB* from the t(5;12)(q31–33;p13) translocation [36,38]. The *ETV6-PDGFRB* fusion gene leads to proliferation mediated by STAT5, NF-κB, and ERK1/2 signaling [39]. Moreover, a number of genes that are important in the proliferation and differentiation of eosinophils are found in the same 5q31–q35 chromosomal region, including interleukin-3, interleukin-4, interleukin-5, interleukin-13, and granulocyte–macrophage colony-stimulating factor. The morphologic features of *PDGFRB*-related disease are variable but are often those of chronic myelomonocytic leukemia, aCML, or chronic myeloproliferative disease with prominent peripheral-blood eosinophilia. Blastic transformation is usually myeloid, and several de novo cases of AML with *ETV6-PDGFRB* and eosinophilia have also been reported. Similar to *FIP1L1-PDGFRA*, eosinophils are often dysmorphic in *ETV6-PDGFRB*. The most common findings are marked hypogranulation (agranular cytoplasm ≥25%), marked cytoplasmic vacuolization (vacuoles ≥25% of cytoplasm), and unilobed nuclei [40]. *PDGFRB* rearrangements are usually easy to detect with cytogenetic analysis; however, some cases require FISH for identification (Figure 7) [41].

#### 3.4.3. AML with *FGFR1* Rearrangements

Fibroblast growth factor receptor 1 (*FGFR1*) rearrangements involving the 8p11 locus were first described in 1992 [42,43]. The most common translocation is t(8;13)(p11;q12), resulting in *ZNF198-FGFR1* [44]. Many other partners have been identified, also resulting in the constitutive activation of FGFR1 tyrosine kinase [45,46,47]. These patients present a wide variety of myeloid and lymphoid neoplasms with peripheral-blood eosinophilia (so-called 8p11 myeloid/lymphoid neoplasms) [48].

Recognizing AML with eosinophilia due to *PDGFRA* and *PDGFRΒ* rearrangement is important, because the aberrant tyrosine kinase activity can make the disease responsive to tyrosine kinase inhibitors such as imatinib. Therefore, we suggest that molecular genetic analysis (FISH and RT-PCR) for *PDGFRA* and *PDGFRΒ* should be carried out in cases of AML with peripheral eosinophilia.

Imatinib can be used effectively to treat patients carrying *PDGFRA* and *PDGFRB* translocations. The dosage ranges from 100 mg (for *PDGFRA* translocations) to 400 mg (for *PDGFRB* translocations) per day. The study by Metzgeroth and colleagues [49] reported encouraging results with imatinib in 17 patients with AML carrying these translocations: complete remission was achieved in all patients and the median overall survival was 88% at 65 months. Complete molecular remissions were achieved after a median of 5 months of imatinib treatment (range: 3–32). Other studies, however, have shown worse outcomes in AML with *PDGFRA* and *PDGFRB* translocations treated with imatinib, particularly in patients with additional cytogenetic (e.g., complex karyotype) or molecular abnormalities, who had a median overall survival of 12.5 months (range, 2–20) [35,50,51,52]. Treatment-emergent imatinib resistance was mediated by mutations in the PDGFRα or PDGFRβ kinase domain. Treatment of AML with *PDGFRA* and *PDGFRB* rearrangement should involve a combination of imatinib plus chemotherapy, with the consideration of allogeneic stem-cell transplantation. In patients with high-grade peripheral eosinophilia, glucocorticoids should be used for 7–10 days in order to avoid rapid eosinophil degranulation and an associated risk of inflammatory cardiac injury after the initiation of imatinib, especially in those with a history of cardiovascular disease and/or elevated cardiac troponin levels [36].

The treatment of AML with *FGFR1* rearrangement is far from standardized, owing to the poor results obtained with present-day therapy. First- and second-generation tyrosine kinase inhibitors have been used with suboptimal results [53]. Recently, pemigatinib, an *FGFR2* inhibitor used in patients with *FGFR2*-rearranged cholangiocarcinoma, received FDA approval for relapsed/refractory myeloid and/or lymphoid neoplasms with *FGFR1* rearrangements. Allogeneic hematopoietic-cell transplantation is indicated for these patients.

### 3.5. Rare Translocations Involved in AML with Eosinophilia

Several other gene rearrangements involving *JAK2* (located on chromosome 9p24) and *FLT3* (located on chromosome 13q12) may rarely manifest as AML with clonal eosinophilia [24]. These *JAK2* translocations (e.g., *PCM1-JAK2*, *ETV6-JAK2*, and *BCR-JAK2*) and *FLT3* translocations (e.g., *ETV6-FLT3*, *BCR-FLT3*, and *FLT3-TRIP11*) are less common than *PDGFRB* translocations, generally manifesting as chronic myeloproliferative disease (e.g., CEL, aCML or CMML with eosinophilia), acute lymphoblastic leukemia, and very rarely as AML with eosinophilia. Cases of AML with *JAK2* and *FLT3* rearrangements are also included in the current WHO classification in the category of “myeloid/lymphoid neoplasms with eosinophilia and tyrosine kinase gene fusions” [24].

These translocations may be identified by conventional cytogenetic analysis, but cryptic rearrangements require FISH or sequencing assays. The presence of +9/+9p chromosomal abnormalities in the context of AML with eosinophilia may be a clue for cryptic *JAK2* structural rearrangements. Cases of AML with *JAK2* rearrangements, e.g., *PCM1-JAK2* resulting from t(8;9)(p22;p24.1), may respond to JAK inhibitors such as ruxolitinib. There have even been reports of treatment-free remission with ruxolitinib [54]. Cases with *FLT3* rearrangement may respond to FLT3 inhibitors. Allogeneic hematopoietic-cell transplantation seems to be the best choice for these patients.

### 3.6. The Authors’ Recommendation for a Practical Approach to AML with Increased Eosinophils

It is not uncommon to find increased marrow eosinophils in patients with AML in the absence of peripheral-blood eosinophilia, particularly in patients with the myelomonocytic subtype. In many cases, this is a non-specific finding occurring in association with non-specific cytogenetic lesions such as 7q deletion or normal karyotypes, but this finding may also indicate recurrent translocations with immediate therapeutic and prognostic relevance, such as *CBFB-MYH11* or *RUNX1-RUNX1T1*. Of note, the likelihood of AML with *CBFB-MYH11* is increased in the presence of abnormal eosinophils containing both eosinophilic and basophilic staining granules. Also, the myeloid blast phase of CML or *BCR-ABL1*-positive AML with an eosinophilic component should always be borne in mind, since about 5% of CML cases are diagnosed in the blast phase without a recognized chronic phase. Obviously, the identification of *BCR-ABL1* has important therapeutic implications. Therefore, we recommend RT-PCR (or FISH) testing for *CBFB-MYH11*, *RUNX1-RUNX1T1*, and *BCR-ABL1* in all cases of AML with increased eosinophils.

If these translocations are negative, there is peripheral eosinophilia, or there is a history of leukocytosis with eosinophilia, we recommend FISH for *ETV6-ABL1*, *FIP1L1-PDGFRA*, *PDGFRB*, or *FGFR1* rearrangements. If FISH testing is negative but clinical suspicion is high, we recommend RT-PCR for *FIP1L1-PDGFRA* and *ETV6-ABL1*. In cases in which none of the tests described above are positive, additional testing with *JAK2* FISH, *FLT3* FISH, and RNAseq for cryptic translocations (if available) may be considered.

## 4. AML with Increased Basophils

In assessing leukemias with a prominent basophilic component, it is important to distinguish whether the basophils constitute a mature or immature population. The best example of the latter is acute basophilic leukemia, a separate subtype of AML in which the primary differentiation is to basophils. AML with t(6;9) is associated with basophilia in 42–62% of cases. Core-binding factor AML can also produce basophilia. Moreover, many myeloid cancers can transform to AML with hyperbasophilia, including CML, aCML, Ph-negative myeloproliferative neoplasms, and myelodysplastic syndromes.

### 4.1. Differential Diagnosis of Leukemias with Basophilic Granules (Table 2)

As mentioned, the presence of blast cells with dark basophilic granules should raise suspicion for acute basophilic leukemia. This condition should always be distinguished from the basophilic myeloid blast crisis of CML (“secondary acute basophilic leukemia”), as well as from AML with *DEK-NUP214* and AML with *RUNX1-RUNX1T1*. It should also be differentiated from acute mast-cell leukemia. Acute promyelocytic leukemia (APL) should always be borne in mind when one sees a case of AML with blasts containing dark granules.

**Table 2:** Differential diagnosis of leukemias with prominent basophilic granules.

Acute basophilic leukemia; Acute mast-cell leukemia; Basophilic blast phase of CML; Blast phase of other MPN, e.g., *JAK2* V617F-positive essential thrombocytosis; AML with t(8;21)(q22;q22); *RUNX1-RUNX1T1*; AML with t(6;9)(p23;q34.1); *DEK-NUP214*; Basophilic subtype of APL (M3b); AML with *PDGRFA-PRKG2* or *PRKG2-PDGFRB* fusion (rare).
Distinguishing features:
Acute basophilic leukemia—MPO^−^, CD123^+^, CD203c^+^, CD9^+^, CD117^−^, tryptase^−^; Mast-cell leukemia—CD117^+^, CD123^−/weak^, CD9^−^, CD25^+/−^, CD2^+/−^, CD203c^−/+^, tryptase^+^, *C-KIT* mutation present; Blast crisis CML—*BCR-ABL1*-positive; Basophilic variant APL—MPO^+^, Auer rods present, *PML-RARα*-positive.

Abbreviations: AML, acute myeloid leukemia; CML, chronic myeloid leukemia; APL, acute promyelocytic leukemia; MPN, myeloproliferative neoplasm.

#### 4.1.1. Basophilic Blast Phase of CML

In most cases of blast-phase CML, the blast lineage is myeloblastic, but may also be monocytic, megakaryocytic, erythroid, eosinophilic, or basophilic (or any combination thereof). Rarely, patients may present in the blast phase with a morphologic picture identical to de novo acute basophilic leukemia. Thus, RT-PCR for *BCR-ABL1* should always be performed in any case of AML with a prominent basophilic component.

Basophils of the blast phase of CML may display dysplastic features such as reduced granulation [55] and tryptase production [56]. The source of the basophilic blast phase of CML may be the CFU-Bas/Eo hybrid progenitor. *IKZF1* mutations that produce either a loss of IKAROS or dominant negative isoforms have been described in CML lymphoblastic crises [57,58]. The disruption of IKAROS activity in primitive CML cells mimics myeloid disease progression with enhanced STAT5 activation and shifted granulopoiesis to the basophilic lineage [59].

#### 4.1.2. AML with t(6;9)(p23;q34.1); *DEK-NUP214*

AML with t(6;9)(p23;q34.1) shows primarily myeloblastic or myelomonocytic differentiation (M2 or M4, according to the FAB classification). It is accompanied by marrow basophilia consisting of mature, dysplastic basophils in 42–62% of cases, and it is often associated with prominent multilineage dysplasia [60]. It is included in the current WHO classification in the category of “acute myeloid leukemia with defining genetic abnormalities” [24].

From a flow-cytometry point of view, the blasts have a non-specific myeloid immunophenotype (MPO^+^, CD34^+^, CD13^+^, CD33^+^, CD117^+^, CD38^+^, CD123^+^, and HLA-DR^+^), whereas basophils can be seen as separate clusters of cells that are positive for CD123, CD33, and CD38 but negative for HLA-DR. TdT expression is common in AML with t(6;9) (50%) [61]. Notably, the presence of *FLT3*-ITD mutations is very common in AML with t(6;9)(p23;q34.1), occurring in approximately 75% of patients [62]. t(6;9)(p23;q34.1) occurs as a sole karyotypic abnormality in most cases, but, rarely, it occurs in association with a complex karyotype. AML with t(6;9)(p23;q34.1) has a poor prognosis, and allogeneic stem cell transplantation is indicated at first complete remission. Given the high frequency of *FLT3*-ITD in AML with t(6;9), patients may benefit from FLT3 inhibitors.

#### 4.1.3. AML with t(8;21)(q22;q22.1); *RUNX1-RUNX1T1*

Core-binding factor AML can produce marrow basophilia: AML with inv(16) or t(16;16) is characterized by the presence of abnormal eosinophils with mixed eosinophilic and basophilic granules, but AML with t(8;21) often has a true basophilic component [60]. In fact, AML with t(8;21) can mimic or masquerade as acute basophilic leukemia [63]. Therefore, RT-PCR (or FISH) testing for *RUNX1-RUNX1T1* should be performed in all cases of AML with prominent basophils.

#### 4.1.4. Acute Mast-Cell Leukemia

**Figure 8:**
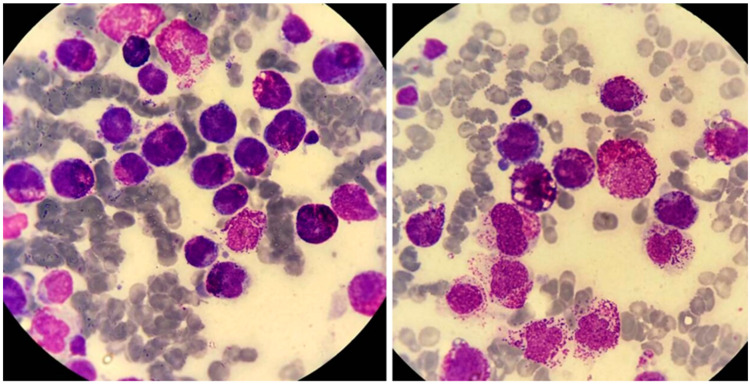
Acute mast-cell leukemia. Bone marrow aspirate smears of a 79-year-old man with aleukemic acute mast-cell leukemia who presented with pancytopenia and abnormal liver function tests. The photomicrographs show round neoplastic cells with dark cytoplasmic granules. The cells were CD13^+^, HLA-DR^+^, CD33^+^, CD203c^+^, CD38^+^, CD2^−^, CD9^−^, CD123^−^, CD11b^−^, FcεRI^+^, CD117^+^, and CD25^+^. PCR was positive for *C-KIT* D816V mutation.

Acute mast-cell leukemia is the leukemic variant of systemic mastocytosis in which peripheral-blood smears contain ≥10% mast cells and bone-marrow aspirates contain ≥20%. A diagnosis of aleukemic mast-cell leukemia is made if the percentage of mast cells in the peripheral-blood smear is <10% (Figure 8) [60]. Unlike in indolent systemic mastocytosis, the mast cells in mast-cell leukemia are often round rather than spindle-shaped. Some of these mast cells may exhibit bilobed nuclei (“promastocytes”). Rare cases, in which the mast cells are mature-appearing and the clinical course less aggressive, constitute chronic mast-cell leukemia.

A strong expression of CD117 and positive staining for tryptase are of great diagnostic value in the diagnosis of acute mast-cell leukemia. Acute mast-cell leukemia may also express myeloid antigens, such as CD13 and CD33, and sometimes, CD203c, CD30, and CD38 [18]. Typically, in systemic mastocytosis, the neoplastic mast cells show a dual expression of CD2 and CD25. In mast-cell leukemia, however, there is often a loss of one or both antigens: a loss of CD25 occurs in 25%, a loss of CD2 in 42%, and 30% of patients are negative for both CD2 and CD25 [64]. The absence of *C-KIT* D816V is more common in CD2- and/or CD25-negative cases [64]. Bone-marrow biopsies shows a diffuse, dense infiltration with atypical mast cells that are tryptase-positive. The majority of patients with mast-cell leukemia have no skin lesions. Acute mast-cell leukemia is an aggressive disease with poor prognosis (median overall survival is ≤12 months). Helpful features in distinguishing patients with acute basophilic leukemia from those with acute mast-cell leukemia are given in Table 3.

#### 4.1.5. Basophilic Variant of APL; *PML-RARA*

The basophilic subtype of APL (subtype M3b, according to the FAB classification) was first described in 1982 as a “hyperbasophilic microgranular variant” of APL [65]. It is characterized by neoplastic promyelocytes with unusual nuclear lobulation and a deeply basophilic cytoplasm containing few or no dark granules. The cytoplasm is scanty and may have cytoplasmic projections or blebs. Like the microgranular subtype of APL, CD2 is positive in the subtype with basophilic cells. In addition, APL with basophil differentiation, with blasts containing large basophilic granules, has been described [19,66]. Aberrations of the 12p13 locus―in addition to t(15;17)(q22;q11-12)―have been described in M3b [19,66]. Relapse of APL after all-trans retinoic acid (ATRA) therapy with M3b morphology may occur. APL with eosinophilic differentiation has also been described. Recognition of the less common, atypical forms of APL with basophilic blasts is important because of the unique response of this disease to retinoic acid and arsenic trioxide.

#### 4.1.6. Acute Basophilic Leukemia

Although acute basophilic leukemia has long been recognized [67], it was not until the 2008 edition of the WHO classification of myeloid neoplasms that it became a distinct clinicopathologic entity classified within the category of AML, not otherwise specified. It is a very rare disease, accounting for <1% of AML cases [60]. It is included in the current WHO classification in the category of “acute myeloid leukemia, defined by differentiation” [24].

**Figure 9:**
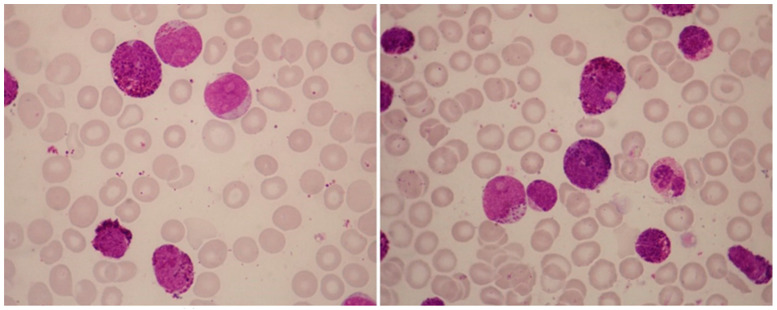
Illustrative case of acute basophilic leukemia (ABL). A 67-year-old man presented for evaluation of general weakness and lower back pain. Over the previous 8 months, he had had postprandial epigastric pain. He had undergone esophagogastroduodenoscopy, which showed the presence of three simultaneous gastric ulcers. Biopsies were negative for *Helicobacter pylori* infection. His past medical history was otherwise unremarkable. He smoked 20 cigarettes daily, drank alcohol rarely, and took no medications. On examination, there was mild hepatomegaly (2 cm below right coastal margin), small-volume peripheral lymphadenopathy (diameter, ≤2 cm), and no splenomegaly. Multiple skin lesions were noted on the trunk and extremities, measuring a few centimeters. They were reddish, reddish brown, or purple, plaque-like, and produced significant itching and discomfort. A complete blood count revealed anemia (hemoglobin, 9.5 g/dL; ΜCV 80 fL), white-cell count 5.1 × 10^9^/L, and platelet count 45 × 10^9^/L. May–Grünwald–Giemsa staining of a peripheral-blood smear revealed 41% neutrophils, 36% lymphocytes, 1% monocytes, 8% nucleated red blood cells, and 22% “atypical” cells with basophilic granules, easily identifiable under oil immersion (×1000). Coagulation studies and hepatic biochemistry were normal, but there was renal dysfunction (urea; 65 mg/dL, creatinine: 2.2 mg/dL) and hypocalcemia (6.2 mg/dL). Lactate dehydrogenase level was 1376 U/L (<246 U/L). Flow cytometry showed that the immature cell population in the CD45^weak^/SSC^low^ gate was CD34^+^, MPO^−^, CD11b^+^, CD13^+^, CD33^+^, CD9^+^, CD123^+^, CD203c^+^, HLA-DR^−^, CD2^−^, CD3^−^, CD5^−^, CD16^−^, CD10^−^, CD22^weak^, CD25^weak^, CD15^−^, TdT^−^, and CD117^−^. An attempted aspiration of the bone marrow yielded a "dry tap”. The most striking feature in this patient was the presence of blast cells with coarse basophilic granules, raising suspicion for ABL. ABL can be identified by expression of either CD123 or CD203c by cells that do not express CD117. The absence of myeloperoxidase (MPO) rules out the possibility of acute promyelocytic leukemia (M3b). In this case, positivity for CD11b and CD123 and the absence of CD117 strongly suggested a diagnosis of ABL. *PML-RARA*, *BCR-ABL1*, *RUNX1-RUNX1T1*, *CBFβ-MYH11*, and *C-KIT* D816V were negative. *NPM1* and *FLT3*-ITD, *FLT3*-TKD mutations were also negative. A peripheral-blood sample for cytogenetic analysis showed no abnormalities (46, XY). The patient’s symptoms can be explained on the basis of hyperhistaminemia. Accordingly, the histamine levels were found to be 710 pg/mL (normal, 0–90 pg/mL). The patient was treated with high doses of two H1 inhibitors, an H2 inhibitor, esomeprazole, and methylprednisone, before initiation of induction chemotherapy.

The characteristic morphologic feature of acute basophilic leukemia is the presence of blasts carrying coarse basophilic granules (see Figure 9 for an example).

According to the current WHO classification [24], there are three diagnostic requirements for acute basophilic leukemia: (1) blasts and mature/immature basophils with metachromatic granules (as shown by toluidine blue stain); (2) blast cytochemistry negative for myeloperoxidase (MPO), Sudan Black B, and non-specific esterase (ANAE); and (3) the absence of strong CD117 expression. Valent and co-workers have proposed simpler diagnostic criteria for acute basophilic leukemia, including the presence of myeloid blasts and metachromatic blasts at ≥20% and basophils at ≥40% of the total nucleated bone-marrow or peripheral-blood cells.

Leukemic cells express antigens of myeloid differentiation such as CD13 and CD33 but are MPO-negative [68,69]. CD34 and HLA-DR show variable expression [70]. Antigens of basophilic differentiation include CD123, CD203c, CD9, IgE receptor (FceRI), and CD11b [71]. Aberrant expression of CD4, TdT, and CD22 has also been reported in acute basophilic leukemia [60,72]. CD25 is usually negative or weakly expressed [68].

On morphological grounds, it is not possible to make a differential diagnosis of acute basophilic leukemia from acute mast-cell leukemia, since blast cells with metachromatic granules are present in both disorders. Specific esterase (chloroacetate esterase [ChlorE]) staining is helpful in distinguishing basophilic from mastocytic granules. It reacts with mastocytic granules but does not react with basophilic granules [73]. Electron microscopy, if available, is also helpful in distinguishing the lineage of the metachromatic blasts: the presence of Θ granules, i.e., electron-dense particles that carry a single fold of their membrane (“theta” character), is typical of basophilic differentiation. In contrast, mast cells carry four different types of granules (crystal-rich, with multiple membrane folds, solid-dense granules, and non-dense granules) without a theta character [69]. The expression of CD123 and/or CD11b indicates basophil differentiation, whereas tryptase and/or CD117 indicate a mast-cell origin. Markers of systemic mastocytosis, i.e., CD2 and CD25, are less helpful [64].

Owing to its rarity, little is known about cytogenetic lesions in acute basophilic leukemia. One abnormality occurring in male infants with acute basophilic leukemia is t(X;6)(p11;q23) [74]. This translocation leads to *MYB-GATA1* fusion, which disrupts the translational regulation by *GATA1*. Since male patients have only one copy of *GATA1* on their X chromosome, *GATA1*-dependent cellular differentiation is completely abrogated. On the other hand, the chimeric protein shows great intracellular stability and retains *MYB* function. The MYB-GATA1 mutant protein is a positive transcriptional regulator of both interleukin 1 receptor-like 1 (*IL1RL1*) and *NTRK1*, which promote basophilic differentiation. Other chromosomal alterations that have been reported include t(16;21)(p11;q22), which creates the *FUS-ERG* fusion gene, and chromosomal aberrations of the 12p13 locus involving *ETV6*, e.g., 12p13 deletion [75,76]. Chromosome 17 abnormalities, as well as mutations in *TP53*, *TET2,* and *NPM1,* have also been reported [77].

Like other AML subtypes, patients with acute basophilic leukemia present with features related to bone-marrow failure and may or may not have circulating blasts. Skin involvement, hepatosplenomegaly, lytic lesions, and symptoms related to hyperhistaminemia may be present. Histamine, an autocrine and paracrine vasoactive hormone, is found in abundance in the metachromatic granules of basophils. Thus, erythematous cutaneous reactions are frequently seen in acute basophilic leukemia. Moreover, histamine promotes hydrochloric acid production in the stomach, causing peptic ulcers and gastritis. Lytic and osteoporotic lesions are also common (histamine affects osteoblasts, inducing RANKL expression, which directly activates osteoclasts [78]). Other symptoms suggestive of hyperhistaminemia include diarrhea, malabsorption, abdominal pain, marked bronchospasm, nausea, and migraine. Induction chemotherapy can worsen or elicit such symptoms as a consequence of the chemotherapy-induced cell lysis and the release of large amounts of histamine in the circulation. Serious complications of hyperhistaminemia during induction chemotherapy include anaphylactic shock, status asthmaticus, pulmonary edema, capillary leak syndrome, arrhythmias, heart failure, gastrointestinal bleeding, and hepatic and coagulation abnormalities [79,80,81,82]. Whilst the anti-leukemic regimens do not differ from standard AML, it is crucial that patients with acute basophilic leukemia be given H1 and H2 inhibitors, proton-pump inhibitors (PPIs), and corticosteroids to abrogate or treat the effects or hyperhistaminemia. The cases observed have generally been associated with a poor prognosis due to both the occurrence of allergic reactions during induction chemotherapy and disease refractoriness.

## 5. Conclusions

In a small percentage of cases of AML, increased eosinophils and/or basophils are present in the cytologic material, usually in the bone marrow and sometimes in the peripheral blood. This is often a puzzling diagnostic situation that requires special investigation with RT-PCR and FISH for specific genetic abnormalities of clinical relevance.

## Figures and Tables

**Table 3:** Acute basophilic leukemia, as compared with acute mast-cell leukemia.

	Acute Basophilic Leukemia	Acute Mast-Cell Leukemia
Clinical features		
Hyperhistaminemia	Common	Common
Skin involvement	Common	Uncommon
Hepatosplenomegaly	Common	Common
Lymphadenopathy	Uncommon	Common
Special stains		
Toluidine Blue	Positive	Positive
Periodic-acid Schiff (P.A.S)	Positive	Negative or weak
Chloracetate esterase (ChorE)	Negative	Positive
Tryptase	Negative or weak	Positive
Myeloperoxidase (MPO)	Negative	Negative
Immunophenotypic studies		
CD34	Negative or weakly positive	Negative or weakly positive
CD45^weak^/CD13/CD33 expression	Positive	Positive
CD14/CD15/CD64 expression	Negative	Negative
CD11b	Positive	Negative
CD17	Positive	Negative
CD123	Positive	Negative
CD203c	Positive	Negative or weakly positive
CD2	Negative	Positive or negative
CD25	Positive	Positive or negative
CD117	Negative	Strongly positive
Cytogenetic and molecular studies		
	t(X;6)(p11;q23)	*C-KIT* mutations (e.g., *C-KIT* D816V)
	t(16;21)(p11;q22)	
	del(12p)	*TET2* mutations
	*TP53*, *TET2* and *NPM1* mutations	*SRSF2*, *ASXL1*, *RUNX1* (“S/A/R”) mutations

## Data Availability

Not applicable.
